# Electrochemical Behaviour of Ti and Ti-6Al-4V Alloy in Phosphate Buffered Saline Solution

**DOI:** 10.3390/ma14247495

**Published:** 2021-12-07

**Authors:** Senka Gudić, Ladislav Vrsalović, Dario Kvrgić, Aleš Nagode

**Affiliations:** 1 Faculty of Chemistry and Technology, University of Split, Ruđera Boškovića 35, 21000 Split, Croatia; senka.gudic@ktf-split.hr (S.G.); dario.kvrgic@gmail.com (D.K.); 2 Faculty of Natural Sciences and Engineering, University of Ljubljana, Aškerčeva Cesta 12, 1000 Ljubljana, Slovenia; Ales.Nagode@ntf.uni-lj.si

**Keywords:** titanium, Ti-6Al-4V alloy, oxide film, phosphate buffer solution, corrosion

## Abstract

The electrochemical behavior of commercially pure titanium (CP Ti) and Ti-6Al-4V (Grade 5) alloy in phosphate buffered saline solution (PBS, pH = 7.4) at 37 °C (i.e., in simulated physiological solution in the human body) was examined using open circuit potential measurements, linear and potentiodynamic polarization and electrochemical impedance spectroscopy methods. After the impedance measurements and after potentiodynamic polarization measurements, the surface of the samples was investigated by scanning electron microscopy, while the elemental composition of oxide film on the surface of each sample was determined by EDS analysis. The electrochemical and corrosion behavior of CP Ti and Ti-6Al-4V alloys is due to forming a two-layer model of surface oxide film, consisting of a thin barrier-type inner layer and a porous outer layer. The inner barrier layer mainly prevents corrosion of CP Ti and Ti-6Al-4V alloy, whose thickness and resistance increase sharply in the first few days of exposure to PBS solution. With longer exposure times to the PBS solution, the structure of the barrier layer subsequently settles, and its resistance increases further. Compared to Ti-6Al-4V alloy, CP Ti shows greater corrosion stability.

## 1. Introduction

Titanium and its alloys possess excellent properties such as low density, good formability, high specific strength and corrosion resistance, weldability and biocompatibility, making them desirable materials, which finds applications in different important areas such as automobile, aerospace, chemical and petrochemical industry, military and medicine [1,2,3,4,5]. Titanium and its alloys are the primary materials for biomedical and dental implant applications. Almost 50% of titanium alloy production refers to Ti-6Al-4V alloy, the most used titanium alloy globally [2]. For biomedical applications, corrosion behavior is one of the most important criteria because the human body is an aqueous electrolyte that contains different ions such as Cl^−^, PO_4_^3−^, HCO_3_^−^, OH^−^, Ca^2+^, Mg^2+^, H^+^, K^+^, Na^+^ etc. [6]. Therefore, many research types were performed to understand better the corrosion behavior of titanium and its alloys in different simulated body fluids [7,8,9,10,11,12,13,14]. The high corrosion resistance of titanium and its alloys is due to the formation of stable and protective oxide layer (mainly consisting of TiO_2_) of a thickness of 2–6 nm. Depending on the environmental conditions, a surface oxide film consists of a two-layer structure: the inner layer is compact, whereas the outer one is porous. The electrochemical properties of the oxide film and its long-term stability in biological environments plays a decisive role in the biocompatibility of titanium implants [10,15,16]. In the case of Ti-6Al-4V alloy, the presence of low contents of aluminium and vanadium oxides (Al_2_O_3_ and V_2_O_5_) in the porous layer of the passive film, have been detected [17,18,19]. These oxides can dissolve and deteriorate the passivity of Ti-6Al-4V alloy. The results of the investigations of Alves et al. [20] have shown the significant influence of the solution temperature and immersion time in corrosion resistance of titanium and Ti-6Al-4V alloys. In Hank’s solution, electrochemical measurements at 25 °C indicate higher corrosion resistance of Ti-6Al-4V alloys compared with pure titanium, which was explained by the beneficial influence of Al and V alloying elements, which reduces the dissolution rate of the passive layer formed in the solution. The same investigations on 37 °C have shown that the passive films have lower resistance on Ti-6Al-4V than that of titanium due to local corrosion processes accelerated by the presence of chloride ions. In addition, corrosion potential changes towards more positive values with time at both temperatures. After corrosion investigations, vanadium and aluminium ions can be found in the electrolyte [20,21,22]. Noumbissi et al. [23], in their review paper, based on 64 scientific articles, points out that corrosion related to titanium and its alloys affects the health of peri-implant soft and hard tissue and the long term survival of metal dental implants.

Gugelmin and associates [24] have investigated the stability of thin titanium dioxide grown by the potentiodynamic method on Ti-6Al-4V surfaces up to 5.0 V in phosphate buffered solution (PBS) and artificial blood media at different immersion times. Electrochemical impedance spectroscopy (EIS) measurements have shown the lower polarization resistance after 30 days of immersion in artificial blood, which is explained by the spontaneous oxide dissolution. This can be originated both from the oxide thickness decrease as the matrix rearrangement occurs during the electrolyte exposure [21,24,25]. In contrast, in PBS solution, there is a significant increase in the polarization resistance with time up to 10 days [24]. Merino and Mascaro [26] have been investigated passive oxide film on Ti-grade 2 dental implant, formed in PBS and its spontaneous dissolution in artificial saliva by EIS measurements over 24 h. Results have shown that the resistance of the passive film on Ti-grade 2 decreases as the exposure time increases due to the breakdown of the oxide followed by a dissolution process. The increase in impedance values with time was also observed for Ti-6Al-4V in Ringer′s solution for the measurement period of 100 h [27]. Results of EIS measurements for Ti-15Mo alloy in PBS solution at 37 °C have shown the increase of the impedance up to 24 h and then decrease up to 360 hours [28]. The initial increase in barrier layer resistance was explained in terms of the oxide film growth, while a slight decrease is a consequence of the chloride ion attack [28]. Zhang and associates have investigated time-dependent corrosion of Ti-6Al-4V in 0.9% NaCl solution in the absence and presence of H_2_O_2_ and albumin. The EIS results have shown that in all solutions, polarization resistance gradually increased with immersion time and subsequently approached a steady-state at around 70 h [29].

Although significant attention was paid to the corrosion testing of Ti and Ti alloys in simulated body solutions, many tests were performed with the short-time corrosion measurements, while the influence of electrolyte exposure time on the corrosion behaviour of Ti and Ti-alloys was investigated in much less extent. In addition, the results obtained in long-term investigations are sometimes contradictory, probably due to different methodologies and investigation conditions.

Therefore, in this work, short-therm and long-therm electrochemical and corrosion behaviour of CP Ti and Ti-6Al-4V alloy in PBS (pH = 7.4) at 37 °C (i.e., in simulated physiological solution in the human body) was examined using open circuit potential measurements, linear and potentiodynamic polarization and electrochemical impedance spectroscopy methods, while the surface condition of the tested samples was examined by SEM/EDS analysis.

## 2. Materials and Methods

Measurements were performed in a standard double wall glass reactor with three electrodes: the working electrode, Pt-sheet counter electrode and saturated calomel reference electrode (SCE) mounted in Luggin capillary. All the potential values are reported herein to the SCE. Investigations were performed on the cylindrical samples of commercial pure titanium, 99.6% purity (CP Ti) and Ti-6Al-4V alloy obtained from Goodfellow Cambridge Ltd. (Huntingdon, UK). The cylindrical samples were joined with insulated copper wire, embedded in polyacrylate to expose only the base of the cylinder to the electrolyte (surface area of 0.2 cm^2^). Before each experiment, the working electrode was abraded mechanically using Metkon Forcipol 1 V grinding and polishing machine (Metkon Instruments Inc., Bursa, Turkey) with successive grades of emery papers up to 2500 grit. Afterwards, the electrode was polished with alumina polishing suspension (0.3 μm), ultrasonically washed with 70% ethanol and Millipore deionized water, and transferred quickly to the electrochemical reactor. The electrochemical behavior of CP Ti and Ti-6Al-4V alloy was investigated in phosphate buffered saline solution (PBS), pH = 7.4, the composition of which is shown in Table 1.

A PBS solution was prepared by dissolving the weighed masses of solid salts (NaCl, KCl, Na_2_HPO_4_ and KH_2_PO_4_) obtained from Sigma (ACS reagent grade) in deionized water, and the pH was adjusted to 7.4. All measurements were performed at a temperature of 37 °C. The electrochemical experiments were performed using a Princeton Applied Research (Princeton, NJ, USA) PAR 273A potentiostat/galvanostat system connected with PAR M 5210 lock-in amplifier for electrochemical impedance spectroscopy measurements.

The electrochemical behaviour of Ti and Ti-6Al-4V alloy in PBS solution was performed by open circuit potential measurements (*E*_OC_) in 60 min time period, linear polarisation method in the potential region of ±20 mV vs. *E*_OC_ with the scan rate of 0.2 mV s^−1^ and potentiodynamic polarisation method in potential range from −0.4 V vs. *E*_OC_ up to 2.0 V with scan rate of 1 mV s^−1^. Linear and potentiodynamic polarization measurements were performed after 60 min stabilization of Ti and Ti-6Al-4V alloy in PBS solution.The influence of stabilization time on the electrical properties of the phase boundary of the tested sample (CP Ti, Ti-6Al-4V)/PBS solution was investigated by electrochemical impedance spectroscopy. For this purpose, the electrode was stabilized on the *E*_OC,_ and impedance spectra were recorded over 14 days. Measurements were performed in the range of 50 kHz to 30 mHz with 5 points per decade and ac voltage amplitude of 10 mV. All electrochemical measurements were performed in triplicate to ensure reproducibility of results.

After potentiodynamic polarization measurements as well as after EIS measurements, surface state and the elemental composition of the individual sample were determined by Field emission scanning electron microscope (FEG SEM, Hillsboro, OR, USA) Thermo Scientific Quattro S with attached EDXS SDD detector Ultim^®^ Max, Oxford Instruments for semiquantitative analysis. For imaging, backscattered (BS) as well as secondary (SE) electrons were used at an accelerating voltage of 15 kV.

## 3. Results and Discussion

**Figure 1:**
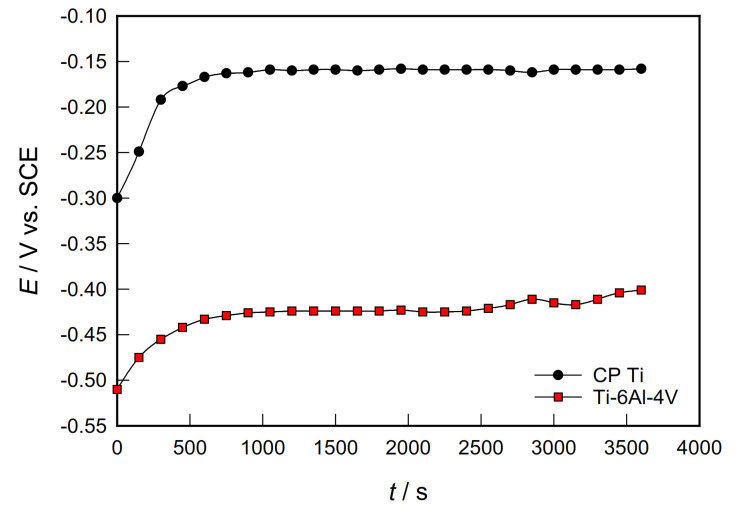
Evolution of open circuit potential over time for investigated samples in PBS solution.

Figure 1 presents the evolution of the *E*_OC_ as a function of time for CP Ti and Ti-6Al-4V samples in PBS solution at 37 °C. *E*_OC_ reflects the composite results of the electrochemical reactions taking place at the electrode/solution interface. 

Both investigated samples showed similar behavior. Namely, immediately after immersion in the PBS solution, the potential of CP Ti and Ti-6Al-4V alloys increases significantly and quickly towards a nobler direction. After only 20 minutes, it establishes a stable value. The potential shift towards more positive values is a consequence of the formation and growth of a protective oxide film on the surface of the tested samples. Compared to the Ti-6Al-4V alloy, *E*_OC_ of CP Ti is even 250 mV more positive. Namely, after one hour, the potential of the open circuit is −150 mV for CP Ti and −400 mV for Ti-6Al-4V alloy. Similar behaviour was observed for Ti and Ti-6Al-4V alloy, both for PBS solution and in other corrosive environments of the human body [8,30,31].

The corrosion behaviour of biocompatible materials in PBS solution was investigated by polarization measurements, i.e., by the method of linear polarization to determine polarization resistance (*R*_p_) and recording potentiodynamic polarization curves in a wide range of potentials for determining corrosion parameters and predicting anodic behavior of Ti (Ti-6V-4Al)/PBS solution systems.

**Figure 2:**
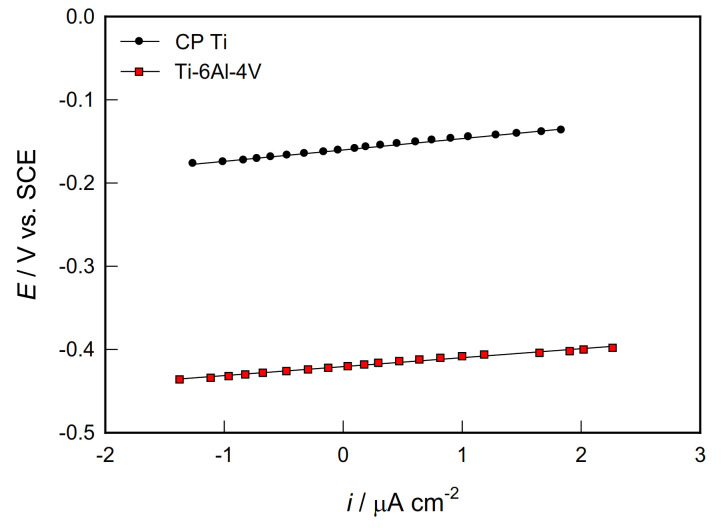
Linear parts of polarization curves for CP Ti and Ti-6Al-4V alloy in PBS solution.

The *R*_p_ data were determined from the slope of linear *i*-*E* dependencies recorded in a narrow potential range ±20 mV vs. *E*_OC_ (Figure 2) and shown in Table 2.

**Figure 3:**
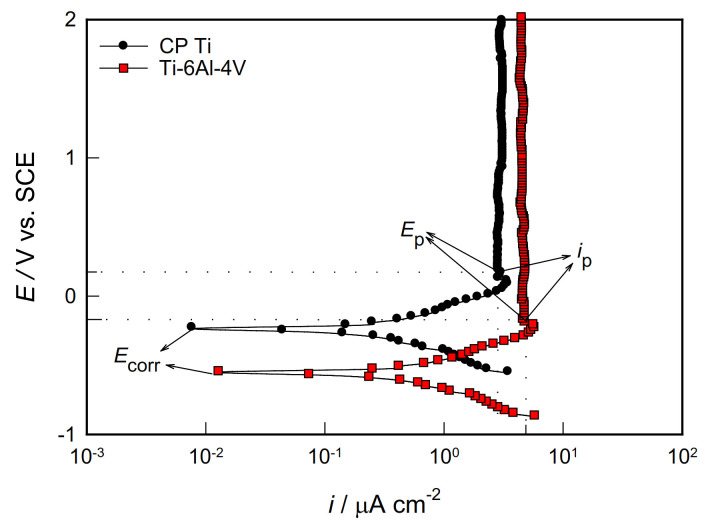
Potentiodynamic polarization curves of the investigated samples in PBS solution.

Performing potentiodynamic polarization measurements is particularly interesting to examine the anodic behavior of a corroding system. The potential of the sample changes slowly in the positive direction, due to which it acts as an anode and corrodes or an oxide layer is formed on it. To investigate the stability of the protective surface oxide film on CP Ti and Ti-6Al-4V alloy at high anodic potentials, potentiodynamic polarization measurements were performed in the potential range from −0.4 V vs. *E*_OC_ up to 2.0 V. The representative polarization curves obtained for both CP Ti and Ti-6Al-4V alloy in the PBS solution is displayed in Figure 3. On the anode branch of polarization curves (potentials more positive than corrosion potential, *E*_corr_), two characteristic areas of potential are observed: active and passive. During the anodic polarization, Ti dissolves in the active potential region and the metal ions (in the form of Ti^4+^) go into solution, and the current increases exponentially with increasing potential (i.e., a linear increase in the logarithm of the anodic current density with potential is observed on the polarization curve). In the electrolyte solution, Ti^4+^ ions come into contact with water and form TiO_2_ film that covers the metal surface and slows down the further process of metal dissolving. With further anodic polarization, the metal dissolution rate significantly slows down by the oxide film formation, which ends at the passivation potential of the metal (*E*_p_) with the passivation current (*i*_p_).

At that moment, the surface of the metal is completely covered with an oxide film, and the current becomes independent of the change in potential, and a “current plateau” is defined on the polarization curve. Namely, “current plateau” (which extends to high anode potentials, up to 2.0 V) is associated with the thickening of the passive film involving some transport process driven the electric field in the oxide layer [32].

As seen from Figure 3, both samples exhibited similar passivation behaviour and even up to 2.0 V the oxide films formed on both samples did not breakdown. Similar behaviour was observed for Ti and Ti-6Al-4V alloy in different corrosive environments of the human body [20,31,33]. For the investigated samples, characteristic values of corrosion potential (*E*_corr_), corrosion current density (*i*_corr_), passivation potential (*E*_p_), and passivation current density (*i*_p_) were determined from the polarization curves and shown in Table 2, along with the data for the polarization resistance (*R*_p_). According to the polarization data, the smaller *i*_corr_ and *i*_p_ and the more positive *E*_corr_ and *E*_p_ showed CP Ti. The CP Ti sample also has a higher value of *R*_p_ (corrosion resistance) due to the better protective properties of the oxide film.

**Table 2:** Corrosion parameters for the CP Ti and Ti-6Al-4V alloy in PBS solution.

Sample	*E*_corr_ (V)	*i*_corr_ (µA cm^−2^)	*E*_p_ (V)	*i*_p_ (µA cm^−2^)	*R*_p_ (kΩ cm^2^)
CP Ti	−0.244 ± 0.012	0.23 ± 0.02	0.151 ± 0.02	2.86 ± 0.014	121.10 ± 8.36
Ti-6Al-4V	−0.569 ± 0.025	0.36 ± 0.03	−0.148 ± 0.034	4.12 ± 0.022	101.22 ± 7.94

**Figure 4:**
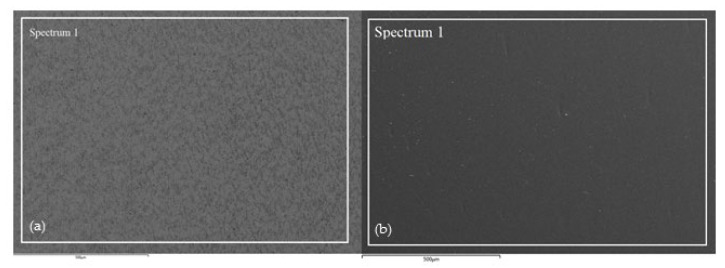
SEM image of (**a**) CP Ti and (**b**) Ti-6Al-4V alloy after potentiodynamic polarization measurements in PBS solution with marked surface of EDS analysis.

After potentiodynamic polarization measurements, the surface condition of CP Ti and Ti-6Al-4V samples was examined by SEM, while the elemental composition of the surface at individual positions was determined by EDS analysis. The obtained results are shown in Figure 4 and Table 3.

**Figure 5:**
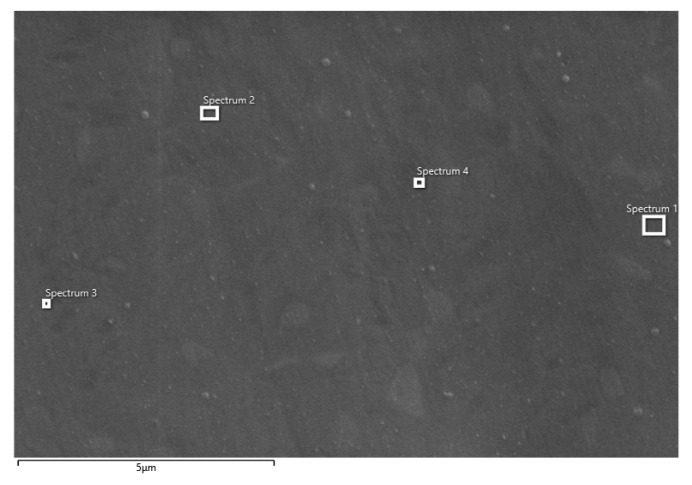
SEM image of Ti-6Al-4V alloy after potentiodynamic polarization measurements in PBS solution with marked spots (position 1–4) of EDS analysis.

EDS analysis of CP Ti shows the presence of Ti and O, while the analysis of Ti-6Al-4V alloy shows the presence of Ti, O, Al, and V (Table 3). The presence of oxygen indicates the fact that the surfaces of both samples are covered with a protective oxide layer. Compared to the Ti-6Al-4V alloy, higher oxygen content was observed on the CP Ti surface, and the oxide layer has a greater thickness. Furthermore, under the influence of anodic polarization on both samples, oxide layers of uniform composition are formed, which is proved by the measurements at high magnifications (Figure 5) and the analysis of surface bends at different positions (Table 4).

**Figure 6:**
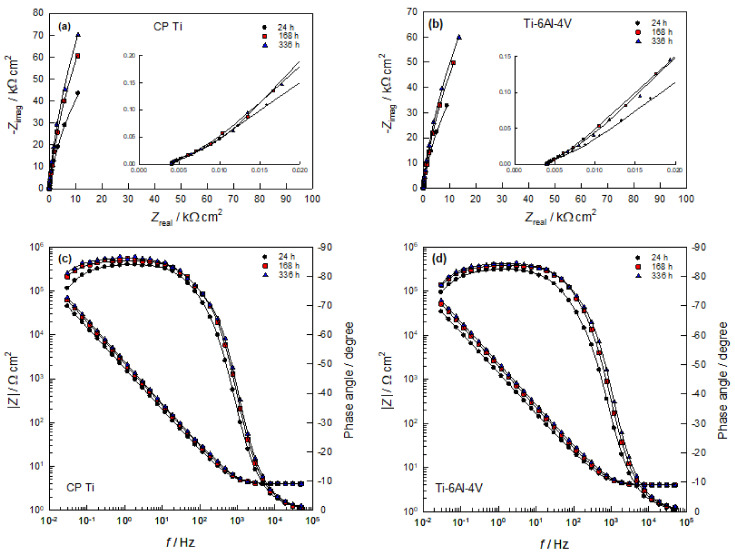
(**a**,**b**) Nyquist (insert: zoomed high-frequency part of impedance spectra) and (**c**,**d**) Bode plots recorded on CP Ti and Ti-6Al-4V alloy in PBS solution at different stabilization times on *E*_OC_.

The electrical properties of the phase boundary of CP Ti (Ti-6Al-4V alloy)/PBS solutions were determined by measuring the impedance at different stabilization times on the *E*_OC_ (up to 14 days), and the obtained results are presented in Nyquist and Bode complex plane (Figure 6). In Figure 6, experimental points are the symbols and the line represents the fitted results. At the first glance, the system’s response in the Nyquist complex plane (Figure 6a,b) for the observed samples is an incomplete capacitive semicircle in the whole frequency range, which indicates that the surfaces of both samples have extremely high resistance (impedance). However, both samples have an additional small impedance loop at high frequencies (which is visible by zoomed of high-frequency part of impedance spectra). The capacitive semicircles are related to the dielectric properties of the naturally formed oxide film on the metals surfaces.

The Bode complex plane (Figure 6c,d) shows the dependences of the absolute value of the impedance and the phase angle on the logarithm of the frequency (log | Z | vs. log *f* and the phase angle vs. log *f*). At high frequencies (*f* > 5 kHz), the influence of electrolyte resistance *R*_el_ is dominant in the total impedance, and the phase shift between current and voltage is ≈ 0°. At medium frequencies (*f* < 1 kHz), the capacitive behavior of the electrode is expressed, which is determined by the dielectric properties of the oxide film (phase angle is ≈90°). This frequency range is determined by the Bode direction with a slope of ≈−1 and extends through the low-frequency range. Compared to the Ti-6Al-4V alloy, the CP Ti sample shows a higher impedance. The impedance of both samples increases with the time of exposure to PBS solution. These results are in accordance with the results of EIS measurements of Fekry and Ameer [27], for the investigation of Ti-6Al-4V alloy in Ringer solution, and with the investigations of Zhang and associates for Ti-6Al-4V alloy in 0.9% NaCl solution [29].

Mathematical analysis showed that the obtained results deviate from the ideal behavior (e.g., Bode’s slope is different from −1) due to inhomogeneities within the oxide layer and the fact that the electrode surface at the microscopic level is not ideally smooth and flat [34,35,36]. Therefore, for the observed frequency range, the electrode impedance, *Z*, is described by a constant phase element (CPE), whose impedance, *Z*_CPE_, is given by the expression [34]:

(1) $$Z_{\mathrm{CPE}}={\left[Q{\left(j\omega \right)}^{n}\right]}^{-1}$$

In the above equation, *j* is an imaginary number (*j* = √−1), *ω* is the circular frequency of the ac signal (*ω* = 2π*f*), *Q* is a frequency-independent constant, and is related to the state of the surface. The exponent of a constant phase element, the magnitude of *n*, is also a constant that can take on different values in the range of −1 to +1. In the case when *n* = 0 the above equation describes the resistance, for *n* = −1 inductance, and for *n* = 1 capacitance.

For the diffusion process through the electrode/electrolyte phase boundary, through the solid phase, and over the entire electrode surface, the magnitude of *n* takes the amount of 0.5 [34].

**Figure 7:**
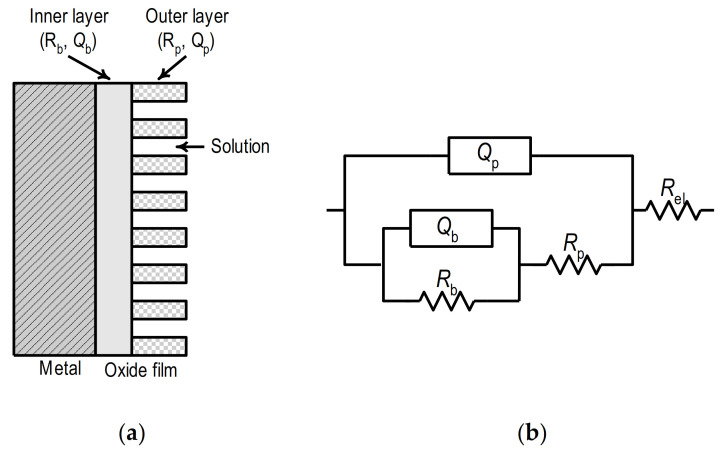
(**a**) Schematic representation of oxide layer on the metal surface, (**b**) equivalent circuit.

The obtained results follow the data given in the literature. It is generally accepted that the oxide film on the surface of Ti and its alloys has a two-layer structure and consists of an inner thin, compact film, so-called barrier film, and outer porous film (Figure 7a) [31,37,38,39]. The inner barrier film has an extremely high impedance, while the outer porous shows significantly lower impedance.

Figure 7b shows the equivalent circuit which was used for analyzing the impedance data. It consists of the electrolyte resistance, *R*_el_ (≈ 5 Ω cm^2^), connected in series with two-time constants. The first and second-time constants are determined by the parallel connection of the constant phase element and the resistance, the first with (*Q*_p_*R*_p_) and the second with (*Q*_b_*R*_b_).

In the presented scheme, the constant phase elements, i.e., the quantities *Q*_p_ and *Q*_b_, represent the capacitances *C*_p_ and *C*_b_ of the oxide film based on the simulation of a certain parameter *n*_p_ and *n*_b_. The time constant, *Q*_p_*R*_p_, observed in the high-frequency range, describes the properties of the porous part of the oxide film. In this case, *R*_p_ is the resistance of the porous film (i.e., the resistance of the electrolyte within the pores), and *Q*_p_ replaces the capacity of the porous film. The time constant in the low-frequency range describes the compact, inner, barrier portion of the oxide film, with *Q*_b_ representing the capacity and *R*_b_ the resistance of the inner barrier film.

By adjusting the measured frequency dependence of the impedance with the theoretical impedance function for the proposed equivalent circuit, the phase boundary parameters of CP Ti (Ti-6Al-4V alloys)/PBS solutions were determined, and the obtained values were given in Table 5. The value of chi-square (*χ*^2^) is very small (≈6 × 10^−4^), illustrating the good quality of the fitted results.

**Figure 8:**
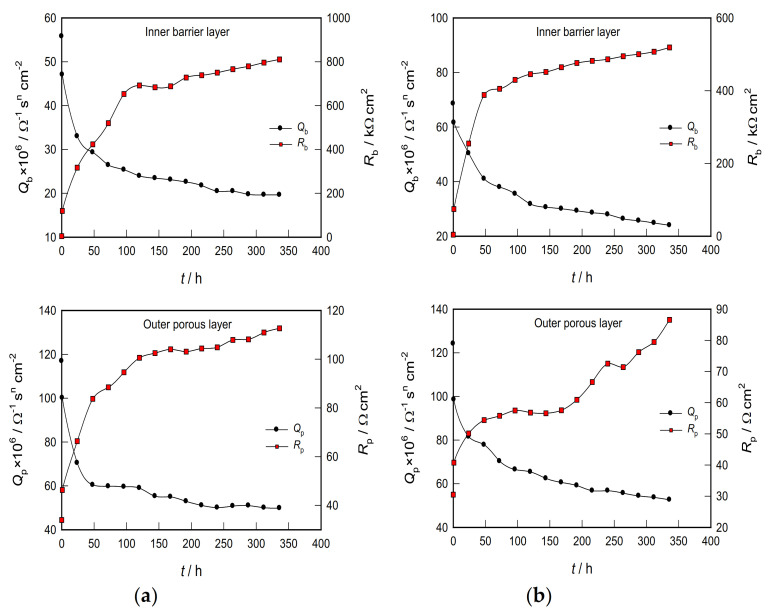
Dependence of the inner barrier layer (*Q*_b_ and *R*_b_) and the outer porous layer (*Q*_p_ and *R*_p_) parameters on the stabilization time for (**a**) CP Ti and (**b**) Ti-6Al-4V alloy in PBS.

To better explain the situation, the time dependences of the parameters depicting the barriers and the porous layer on the CP Ti and Ti-6Al-4V alloys were observed separately (Figure 8).

As shown in Figure 8a, the resistance of the barrier film on CP Ti is exceptionally high. It increases with exposure time, especially in the first five days, where an increase of ≈5 kΩ cm^2^ (immediately after immersion in the solution) to ≈700 kΩ cm^2^ (after five days) was observed. Further exposure of the sample to PBS solution shows a slight increase in the resistance of the barrier film, and after 14 days it values is ≈800 kΩ cm^2^. In addition, the capacity of the barrier film is relatively tiny and decreases slightly with time (somewhat faster in the first three days) and, after 14 days, reaches a stationary value of ≈19 × 10^−6^ Ω^−1^ s^n^ cm^−2^.

According to the plate capacitor model, the capacity is inversely proportional to the thickness, *d* (*C* = *ε ε*_o_/*d*; where *ε*_o_ is the dielectric constant of vacuum (8.85 × 10^−12^ F m^−1^), and *ε* is the dielectric constant of the TiO_2_ film (≈100) [40]. Therefore, the decrease in the size of *Q* (from ≈55 to ≈19 × 10^−6^ Ω^−1^ s^n^ cm^−2^) with increasing time corresponds to an adequate increase in the thickness of the barrier part of the oxide layer.

On the other hand, the resistance of the porous layer on CP Ti is relatively small and increases with the exposure time of the metal to the electrolyte (roughly from 35 to 110 Ω cm^2^). This indicates the fact that the pores of the oxide film are most likely filled with an electrolyte solution. The capacity of the porous film after 14 days of exposure is ≈50 × 10^−6^ Ω^−1^ s^n^ cm^−2^. Due to the open structure, it is difficult to determine the thickness of the outer porous layer [41]. Based on the above, the corrosion of CP Ti is mainly prevented by the inner barrier layer, whose thickness and resistance increase sharply in the first few days of exposure to PBS solution, after which the thickness remains approximately constant and a subsequent arrangement of the structure further increases the film resistance.

The film on Ti-6Al-4V alloy behaves similarly (Figure 8b), with a minor exception relating to the porous part of the oxide layer. Namely, it was noticed that the resistance of the porous layer increases sharply with prolonged exposure to the electrolyte solution. In addition, from Figure 8, it is clearly seen that the oxide film (barrier and porous part) on CP Ti has a higher resistance compared to the film formed in PBS solution on Ti-6Al-4V alloy. The CP Ti sample also has a lower barrier and porous film capacity value (i.e., greater thickness of both films), indicating better protective properties of the surface film on CP Ti than Ti-6Al-4V alloy.

**Figure 9:**
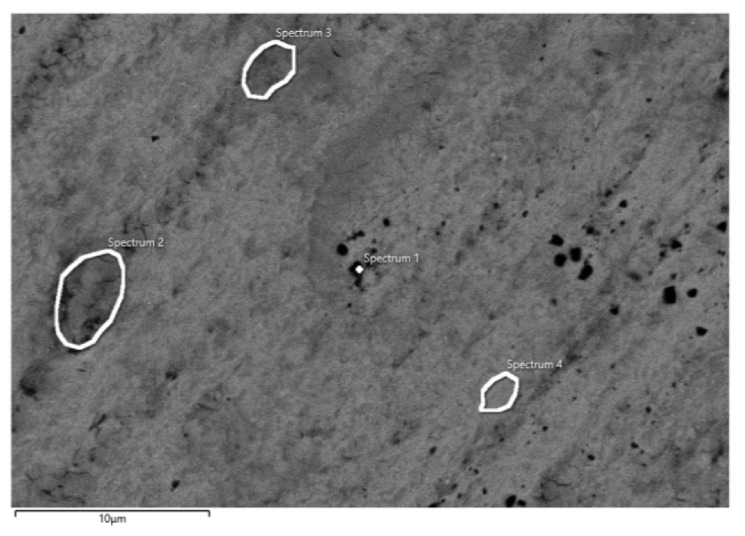
SEM image of CP Ti after impedance measurements in PBS solution with marked spots of EDS analysis.

**Figure 10:**
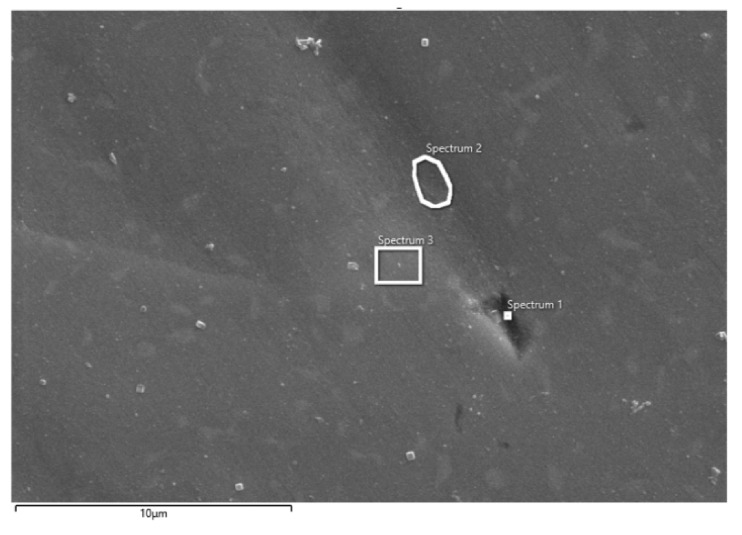
SEM image of Ti-6Al-4V alloy after impedance measurements in PBS solution with marked spots of EDS analysis.

After impedance measurements, the surface condition of CP Ti and Ti-6Al-4V samples were also examined by SEM/EDS analysis and the obtained results are shown in Figure 9 and Figure 10 and Table 6 and Table 7. Since backscattered electrons that are atomic sensitive were used for the imaging, the number of electrons backscattered from a specimen increases with the average atomic number of the specimen. The darker spots thus represent the lower average atomic number region of the specimen. On the other hand, the secondary electrons provide information on surface topography. Anyway, the areas where the elementary composition was measured are marked in Figures, and the obtained results (for different positions) are listed in the corresponding Tables.

EDS analysis of CP Ti (Table 6) shows the presence of Ti and O at varying amounts at positions 1–4, which indicates the fact that the entire surface of CP Ti is covered with a protective oxide layer of TiO_2_ [6,10,17,20,41]. As already established, the oxide layer has a two-layer structure, and the outer part of the oxide layer is porous in nature. Namely, in some places of the surface (positions 1 and 2) EDS analysis revealed the presence of Na and P, which were incorporated from the electrolyte, probably into the pores of the outer part of the oxide layer. Furthermore, aggressive Cl- ions were not observed on the Ti surface, and thus, the oxide film was undamaged.

**Table 6:** Elemental composition on different spots (position 1–4) on surface CP Ti after impedance measurements in PBS solution.

Element	Spectrum 1	Spectrum 2	Spectrum 3	Spectrum 4
O	8.59	14.11	14.13	5.72
Na	0.09	0.14	-	-
P	0.08	0.09	-	-
Ca	-	0.22	0.19	-
Ti	91.24	85.44	85.68	94.28
Total	100.00	100.00	100.00	100.00

EDS analysis of Ti-6Al-4V alloy (Table 7) confirmed the expected presence of Ti, Al V and O in different percentages depending on the observed surface location (positions 1–3). An extremely high percentage of oxygen was observed at position 1, which indicates the fact that the entire surface of the alloy is covered with a rather non-uniform oxide layer. Furthermore, in positions 1 and 2, certain content of Cl^−^ ions was observed, which implies the possible occurrence of damage in the oxide layer. In addition to the above, over the entire surface (positions 1–3), EDS analysis revealed the presence of P (and Na, position 1), which was probably incorporated from the electrolyte into the pores of the outer part of the oxide. Compared to CP Ti, a higher component content from the PBS solution is incorporated into the oxide layer of Ti alloy.

**Table 7:** Elemental composition of Ti-6Al-4V alloy on different spots (position 1–3) on surface Ti-6Al-4V alloy after impedance measurements in PBS solution.

Element	Spectrum 1	Spectrum 2	Spectrum 3
O	28.89	3.78	2.79
Na	0.26	-	-
Al	11.52	5.68	6.66
P	0.18	0.05	0.01
Cl	0.07	0.02	-
Ca	0.12	-	-
Ti	57.01	83.97	86.80
V	1.95	6.50	3.75
Total	100.00	100.00	100.00

The EIS results obtained indicate that a subsequent increase of the thickness and resistance (ordering of the structure) of natural oxide layers occurs during long-term stabilization of Ti and its alloy at the open circuit potential, which implies better corrosion stability of these biomaterials in the biological fluid.

Although the indigenous passive oxide film suppresses the Ti and Ti alloy corrosion to promote biocompatibility, the major disadvantage of Ti surfaces is their continuous depassivation/repassivation under natural mechanical stress in body fluids [42,43,44]. These two competing processes can lead to the incorporation of different alloy elements and surrounding solutions into the passive film [16,17,42,44], which is even in static conditions confirmed in this paper by EDS analysis (due to the poorer corrosion stability of the alloy, the content of components that are incorporated from the PBS solution into its oxide layer is also higher). These alloying elements and impurities are most certainly not involved in significant content. Still, dissolution of alloying elements and incorporation of different elements from surrounding solutions into the passive film when the implant is present in the body are possible. These effects may play a role in orthopedic implants since repassivation of Ti at the osseous implantation site leads to the adsorption of calcium and phosphate ions into the passive film [42,44].

Taking this into account, the need arises for suitable surface modification of Ti and its alloys that will result in improved biocompatibility and osteointegration, with simultaneous reduction of strong bacterial seeding on the implant surface [42,44,45,46,47,48], which will be considered through the future research. The starting point in this direction of research is that the anodic polarization of CP Ti and Ti-6Al-4V alloy (in PBS, and probably other suitable electrolytes) leads to surface modification with the formation of oxide films with uniform composition. As can be seen, the anodic oxide films on CP Ti and Ti-6Al-4V alloy consist only of base metal components and oxygen (Table 3 and Table 4), and the electric field during anodic polarization does not draw electrolyte components into the oxide layer. It was also observed that the anodic oxide films were thicker at CP Ti (higher oxygen content) than at Ti-6Al-4V alloy. On the other hand, natural oxide films formed by long-term exposure of CP Ti and Ti-6Al-4V alloy to PBS solution by a slow depassivation/repassivation process can draw electrolyte components into the oxide layer (Table 6 and Table 7).

## 4. Conclusions

The electrochemical behavior of biocompatible CP Ti and Ti-6Al-4V alloy materials in PBS solution at 37 °C was studied. The analysis of the obtained results established that: 

Immediately after immersion in PBS solution, the potential of CP Ti and Ti-6Al-4V alloys increases significantly and very quickly; after only 20 min, it establishes a stable value. Compared to Ti-6Al-4V alloy, the potential of pure CP Ti open circuit is even 250 mV more positive;

The corrosion resistance of CP Ti is significantly higher than the resistance of the Ti-6Al-4V alloy. Namely, CP Ti has a lower corrosion current and passivation current and a higher polarization resistance;

Anode oxide films on CP Ti and Ti-6-Al-4V alloy have a uniform composition (consisting of base metal components and oxygen);

The resistance of CP Ti and Ti-6Al-4V alloys is a consequence of forming a surface oxide layer that has a two-layer structure and consists of an inner barrier and an outer porous film. The inner barrier film has an extremely high resistance, while the outer porous film shows significantly lower resistance, and its pores are mostly filled with electrolyte solution (confirmed by EDS surface analysis);

The inner barrier layer prevents corrosion of CP Ti and Ti-6Al-4V alloys, whose thickness and resistance increase sharply in the first few days of exposure to PBS solution. The film resistance is further increased by subsequent adjustment of the structure.

## Figures and Tables

**Table 1:** The composition of phosphate buffered saline solution.

Salt	Concentration (mmol L^−1^)	Concentration (g L^−1^)
NaCl	137	8.0
KCl	2.7	0.2
Na_2_HPO_4_	10	1.42
KH_2_PO_4_	1.8	0.24

**Table 3:** Elemental composition on marked surface on surface CP Ti and Ti-6Al-4V alloy after potentiodynamic polarization measurements in PBS solution.

	CP Ti	Ti-6Al-4V
Element	Spectrum 1	Spectrum 1
O	7.12	4.21
Ti	92.88	86.51
Al	-	5.12
V	-	3.85
Fe	-	0.31
Total	100.00	100.00

**Table 4:** Elemental composition on different spots (position 1–4) on surface Ti-6Al-4V alloy after potentiodynamic polarization measurements in PBS solution.

Element	Spectrum 1	Spectrum 2	Spectrum 3	Spectrum 3
O	3.59	4.04	3.77	3.68
Al	5.29	5.60	5.22	5.23
Ti	87.88	87.34	86.96	87.62
V	3.25	3.01	4.04	3.48
Total	100.00	100.00	100.00	100.00

**Table 5:** Electrical parameters of equivalent circuit obtained by fitting the experimental results of EIS for CP Ti and Ti-6Al-4V alloy in PBS solution at different stabilization times on *E*_OC_.

t (h)	Q_p_ × 10^6^ (Ω^−1^ s^n^ cm^−2^)	n_p_	R_p_ (Ω cm^2^)	Q_b_ × 10^6^ (Ω^−1^ s^n^ cm^−2^)	n_b_	R_b_ (kΩ cm^2^)
**CP Ti**						
0	116.91 ± 2.59	0.92 ± 0.02	34.01 ± 2.83	55.77 ± 3.06	0.84 ± 0.02	4.64 ± 0.23
1	100.11 ± 3.20	0.96 ± 0.01	46.31 ± 2.54	47.05 ± 2.74	0.86 ± 0.01	119.63 ± 12.35
24	70.45 ± 3.57	0.97 ± 0.01	66.34 ± 3.14	32.99 ± 2.22	0.86 ± 0.01	316.83 ± 22.87
48	60.41 ± 3.80	0.96 ± 0.02	83.72 ± 3.39	29.33 ± 1.09	0.86 ± 0.01	423.20 ± 22.85
72	59.76 ± 2.31	0.96± 0.01	88.45 ± 3.87	26.42 ± 1.05	0.85 ± 0.02	519.77 ± 14.55
96	59.54 ± 2.57	0.96 ± 0.01	94.63 ± 3.53	25.37 ± 1.42	0.87 ± 0.01	652.71 ± 25.28
120	58.99 ± 2.33	0.97 ± 0.01	100.57 ± 4.01	23.95 ± 0.87	0.87 ± 0.01	691.76 ± 25.37
144	55.28 ± 3.39	0.97 ± 0.01	102.50 ± 3.79	23.44 ± 1.21	0.87 ± 0.01	683.55 ± 32.14
168	54.96 ± 2.45	0.98 ± 0.01	104.06 ± 3.77	23.09 ± 0.85	0.88 ± 0.03	688.15 ± 35.23
192	52.92 ± 3.42	0.97 ± 0.02	103.11 ± 3.83	22.58 ± 0.62	0.87 ± 0.02	728.04 ± 19.38
216	51.09 ± 2.39	0.98 ± 0.01	104.40 ± 3.90	21.76 ± 0.81	0.88 ± 0.01	738.44 ± 20.68
240	50.07 ± 2.44	0.97 ± 0.01	104.83 ± 3.95	20.47 ± 1.76	0.89 ± 0.01	750.19 ± 21.01
264	50.77 ± 2.41	0.98 ± 0.01	107.90 ± 3.13	20.48 ± 0.77	0.89 ± 0.01	766.04 ± 24.45
288	51.00 ± 2.42	0.98 ± 0.01	108.12 ± 3.97	19.74 ± 1.13	0.90 ± 0.01	778.99 ± 22.81
312	50.03 ± 2.43	0.98 ± 0.01	110.94 ± 2.13	19.65 ± 1.20	0.91 ± 0.01	796.16 ± 14.29
336	49.84 ± 2.19	0.98 ± 0.01	112.68 ± 1.92	19.63 ± 1.14	0.91 ± 0.02	810.66 ± 12.69
**Ti-6Al-4V**						
0	124.20 ± 3.10	0.90 ± 0.01	30.52 ± 2.59	68.58 ± 3.12	0.82 ± 0.03	4.24 ± 0.57
1	98.54 ± 3.49	0.95 ± 0.02	40.76 ± 2.74	61.63 ± 2.77	0.84 ± 0.02	74.70 ± 11.07
24	81.51 ± 5.21	0.96 ± 0.01	50.14 ± 2.94	50.38 ± 2.83	0.85 ± 0.01	254.81 ± 12.42
48	77.81 ± 4.21	0.95 ± 0.01	54.40 ± 2.58	41.04 ± 2.08	0.84 ± 0.01	388.13 ± 15.33
72	70.31 ± 4.87	0.96 ± 0.01	55.81 ± 2.50	37.97 ± 1.07	0.85 ± 0.01	405.24 ± 17.34
96	66.50 ± 3.55	0.96 ± 0.01	57.52 ± 1.52	35.51 ± 2.34	0.84 ± 0.01	429.52 ± 12.13
120	65.41 ± 3.76	0.96 ± 0.01	56.84 ± 2.61	31.77 ± 2.77	0.85 ± 0.01	445.42 ± 12.83
144	62.44 ± 3.67	0.97 ± 0.01	56.60 ± 2.59	30.60 ± 1.72	0.86 ± 0.01	451.38 ± 12.57
168	60.58 ± 3.88	0.97 ± 0.01	57.55 ± 2.80	30.04 ± 1.75	0.85 ± 0.02	464.35 ± 11.84
192	59.16 ± 3.71	0.96 ± 0.01	60.93 ± 3.32	29.30 ± 0.99	0.86 ± 0.01	476.00 ± 12.39
216	56.79 ± 3.42	0.96 ± 0.02	66.62 ± 2.60	28.59 ± 0.95	0.86 ± 0.01	482.02 ± 13.48
240	56.79 ± 3.89	0.97 ± 0.01	72.51 ± 1.58	27.96 ± 1.22	0.86 ± 0.02	486.35 ± 13.22
264	55.68 ± 3.57	0.98 ± 0.01	71.40 ± 4.22	26.36 ± 1.06	0.87 ± 0.01	494.58 ± 12.86
288	54.42 ± 3.66	0.97 ± 0.01	76.23 ± 3.58	25.66 ± 1.24	0.88 ± 0.01	500.24 ± 12.45
312	53.72± 4.19	0.98 ± 0.01	79.47 ± 2.95	24.82 ± 1.39	0.88 ± 0.01	507.23 ± 12.99
336	52.63 ± 3.41	0.97 ± 0.01	86.58 ± 1.80	23.97 ± 1.36	0.87 ± 0.02	518.72 ± 13.30

## Data Availability

The data presented in this study are available on request from the corresponding author.
